# HIV-1 Transmission Patterns Within and Between Risk Groups in Coastal Kenya

**DOI:** 10.1038/s41598-020-63731-z

**Published:** 2020-04-21

**Authors:** George M. Nduva, Amin S. Hassan, Jamirah Nazziwa, Susan M. Graham, Joakim Esbjörnsson, Eduard J. Sanders

**Affiliations:** 1 Lund University, Lund, Sweden; 2 KEMRI/Wellcome Trust Research Programme, Kilifi, Kenya; 3 University of Washington, Seattle, WA USA; 4 The University of Oxford, Oxford, United Kingdom

**Keywords:** Molecular evolution, HIV infections

## Abstract

HIV-1 transmission patterns within and between populations at different risk of HIV-1 acquisition in Kenya are not well understood. We investigated HIV-1 transmission networks in men who have sex with men (MSM), injecting drug users (IDU), female sex workers (FSW) and heterosexuals (HET) in coastal Kenya. We used maximum-likelihood and Bayesian phylogenetics to analyse new (N = 163) and previously published (N = 495) HIV-1 polymerase sequences collected during 2005–2019. Of the 658 sequences, 131 (20%) were from MSM, 58 (9%) IDU, 109 (17%) FSW, and 360 (55%) HET. Overall, 206 (31%) sequences formed 61 clusters. Most clusters (85%) consisted of sequences from the same risk group, suggesting frequent within-group transmission. The remaining clusters were mixed between HET/MSM (7%), HET/FSW (5%), and MSM/FSW (3%) sequences. One large IDU-exclusive cluster was found, indicating an independent sub-epidemic among this group. Phylodynamic analysis of this cluster revealed a steady increase in HIV-1 infections among IDU since the estimated origin of the cluster in 1987. Our results suggest mixing between high-risk groups and heterosexual populations and could be relevant for the development of targeted HIV-1 prevention programmes in coastal Kenya.

## Introduction

Approximately 5.6% in the population in Kenya are infected by HIV-1, with a more than three-fold higher HIV-1 prevalence among so-called high-risk groups – including men who have sex with men (MSM), injecting drug users (IDU) and female sex workers^1–4^. Modelling data on modes of HIV-1 transmission in Kenya have shown that at least one-third of all new infections occur among high-risk groups^5^. However, little is known about local HIV-1 networks and transmission within and between high-risk groups and the heterosexual (HET) population in African settings^6^. Molecular epidemiology studies in coastal Kenya have described a dynamic HIV-1 epidemic characterised by subtypes A, C, D, and different circulating recombinant forms (CRFs)^6–15^. These studies have indicated high proportions of recombinants within HET, but this was not evident among MSM in a recent study^8^, alluding to separate epidemics. One study in coastal Kenya observed similar HIV-1 recombination patterns among HIV-1 strains in MSM and FSW, suggesting reinfections within mixed networks^13^.

HIV-1 transmission dynamics can be assessed by linking socio-demographic, clinical and behavioural data with HIV-1 sequence data by phylogenetics^16,17^. With few exceptions, most phylogenetic studies of the HIV-1 epidemic in sub-Saharan Africa have focused on understanding HIV-1 subtype diversity and prevalence of antiretroviral resistance mutations^18–24^. Phylogenetic studies highlighting the dynamics of HIV-1 transmission and contribution of high-risk groups to onward viral transmission are common in North America and Europe, where largescale HIV-1 sequence data are available^25–32^. Due to the limited availability of HIV-1 sequences from sub-Saharan Africa, only a few phylogenetic studies have assessed the dynamics of the HIV-1 epidemic in the region^33–35^. Transmission networks studies in Kenya have demonstrated clustering of MSM sequences with evidence of transmission between different geographical regions, and limited mixing between MSM and HET^6,8,13^. We have also demonstrated extensive clustering of HIV-1 *pol* sequences from MSM who have sex with only men and MSM who have sex with both men and women in coastal Kenya^8^. Given that many MSM on the coast of Kenya have sex with both men and women, there is a possibility of HIV-1 transmission linkages between MSM and the local HET community^4^. The primary objective of the current study was to investigate transmission networks within and between MSM, IDU, FSW, and HET in coastal Kenya using both newly generated and previously published HIV-1 *pol* sequences. A secondary objective was to determine HIV-1 genetic diversity among different risk groups in coastal Kenya.

## Results

### Study Population, sequence dataset and sampling density

The analysed 658 coastal Kenyan HIV-1 partial *pol* sequences included both newly generated (N = 163) and previously published sequences (N = 495). Sequences were collected during 2005–2019 in the Mombasa (N = 210, 32%) and Kilifi (N = 448, 68%) counties in coastal Kenya (Table 1). The risk groups included MSM (N = 131, 20%), IDU (N = 58, 9%), FSW (N = 109, 17%), and HET (N = 360, 55%). Based on size estimation of risk groups and the number of infected populations infected with HIV-1 in Mombasa and Kilifi counties^36^, our study was more powered to pick out MSM (sampling density, 51%) and IDU (sampling density, 12%) clusters compared with FSW (sampling density 3%) and HET (sampling density, 1%) clusters (Supplementary Table S1).

**Table 1:** Demographics and distribution of newly generated and published coastal Kenya HIV-1 *pol* sequences by risk-group.

Risk group		MSM (N = 131, 20%)	IDU (N = 58, 9%)	FSW (N = 109, 17%)	HET (N = 360, 55%)	Total (N = 658, 100%)
Sequences	New	9 (7%)	0 (0%)	102 (94%)	52 (14%)	163 (25%)
	Published	122 (93%)	58 (100%)	7 (6%)	308 (86%)	495 (75%)
Subtype	A	92 (70%)	51 (88%)	71 (65%)	217 (60%)	431 (66%)
	C	9 (7%)	2 (3%)	9 (8%)	26 (7%)	46 (7%)
	D	13 (10%)	5 (9%)	12 (11%)	39 (11%)	69 (10%)
	Others^*^	17 (13%)	0 (0%)	17 (16%)	78 (22%)	112 (17%)
Year (range)	2005–2007	27 (21%)	0 (0%)	54 (50%)	24 (7%)	105 (16%)
	2008–2010	60 (46%)	58 (100%)	41 (38%)	302 (84%)	461 (70%)
	2011–2013	18 (14%)	0 (0%)	0 (0%)	0 (0%)	18 (3%)
	2014–2016	14 (11%)	0 (0%)	10 (9%)	32 (9%)	56 (8%)
	2017–2019	12 (9%)	0 (0%)	4 (4%)	2 (1%)	18 (3%)
Area	Mombasa county	74 (57%)	58 (100%)	7 (6%)	71 (20%)	210 (32%)
	Kilifi county	57 (44%)	0 (0%)	102 (94%)	289 (80%)	448 (68%)

Abbreviations: MSM, men who have sex with men; IDU, injecting drug user; FSW, female sex worker; HET, at-risk men and women who did not report sex work or male same-sex behaviour.

^*^Subtype/recombinant (N, %): B (1, 0.2%), G (2, 0.5%), A1D (42, 6.4%), A1C (18, 2.7%), A2D (13, 2.0%), 16_A2D (10, 1.5%), A2_16A2D (6, 0.9%), CD (6, 0.9%), A1A2 (3, 0.5%), A1A2D (3, 0.5%), A1_16A2D (1, 0.2%), A1A2_16A2D (1, 0.2%), A1A2C (1, 0.2%), A1A2CD (1, 0.2%), CA1D (1, 0.2%), DG (1, 0.2%).

### HIV-1 subtypes A, C, and D dominated the epidemic in coastal Kenya

Phylogenetic analysis was used to determine the subtype distribution in the full coastal Kenya sequence dataset (N = 658). In total, 431 subtype A (66%), 46 subtype C (7%), 69 subtype D (10%), 2 subtype G (>1%), and 110 CRF and unique recombinant form (URFs, 17%) sequences were identified (Table 1 and Supplementary Fig. S1). In addition, all subtype A sequences belonged to sub-subtype A1. Detailed recombination analyses of newly generated sequences demonstrated extensive recombination between subtypes, sub-subtypes, and recombinant forms (Supplementary Table S2).

### Identification of coastal Kenya-specific HIV-1 transmission clusters

Maximum-likelihood (ML) phylogenies were reconstructed independently for the most prevalent HIV-1 subtypes in the population (subtypes A, C, and D). Reference sequences were obtained from GenBank based on similarity (whereof 731 participant-unique sub-subtype A1 sequences remained after removal of redundancies; 256 for subtype C; and 92 for subtype D).

Transmission networks were classified based on the number of sequences per cluster into dyads (2 sequences), networks (3–14 sequences), and large clusters (>14). Of the 658 coastal Kenyan sequences, 206 sequences (31%) formed 61 statistically supported clusters (size range: 2–41 sequences). These included 39 dyads (64% of all clusters), 21 networks (34%), and one large cluster (2%) (Table 2 and Supplementary Table S3). Most of the clusters were found among the subtype A sequences (N = 50, 82%), followed by the subtype D sequences (N = 7, 11%), and the subtype C sequences (N = 4, 7%) (Supplementary Fig. S2).

**Table 2:** The number of coastal Kenyan transmission clusters by cluster size and risk group.

Risk group	Dyads (2 sequences, N = 39, 64%)	Networks (3–14, N = 21, 34%)	Large clusters (≥14, N = 1, 2%)	Total clusters (N = 61, 100%)
MSM	11	13	0	24 (39%)
IDU	3	0	1	4 (7%)
FSW	6	0	0	6 (10%)
HET	13	5	0	18 (30%)
HET/FSW	2	1	0	3 (5%)
MSM/HET	2	2	0	4 (7%)
MSM/FSW	2	0	0	2 (3%)

Abbreviations: MSM, men who have sex with men; IDU, injecting drug user; FSW, female sex worker; HET, at-risk men and women who did not report sex work or male same-sex behaviour.

### Risk-group specific clustering patterns

**Figure 1:**
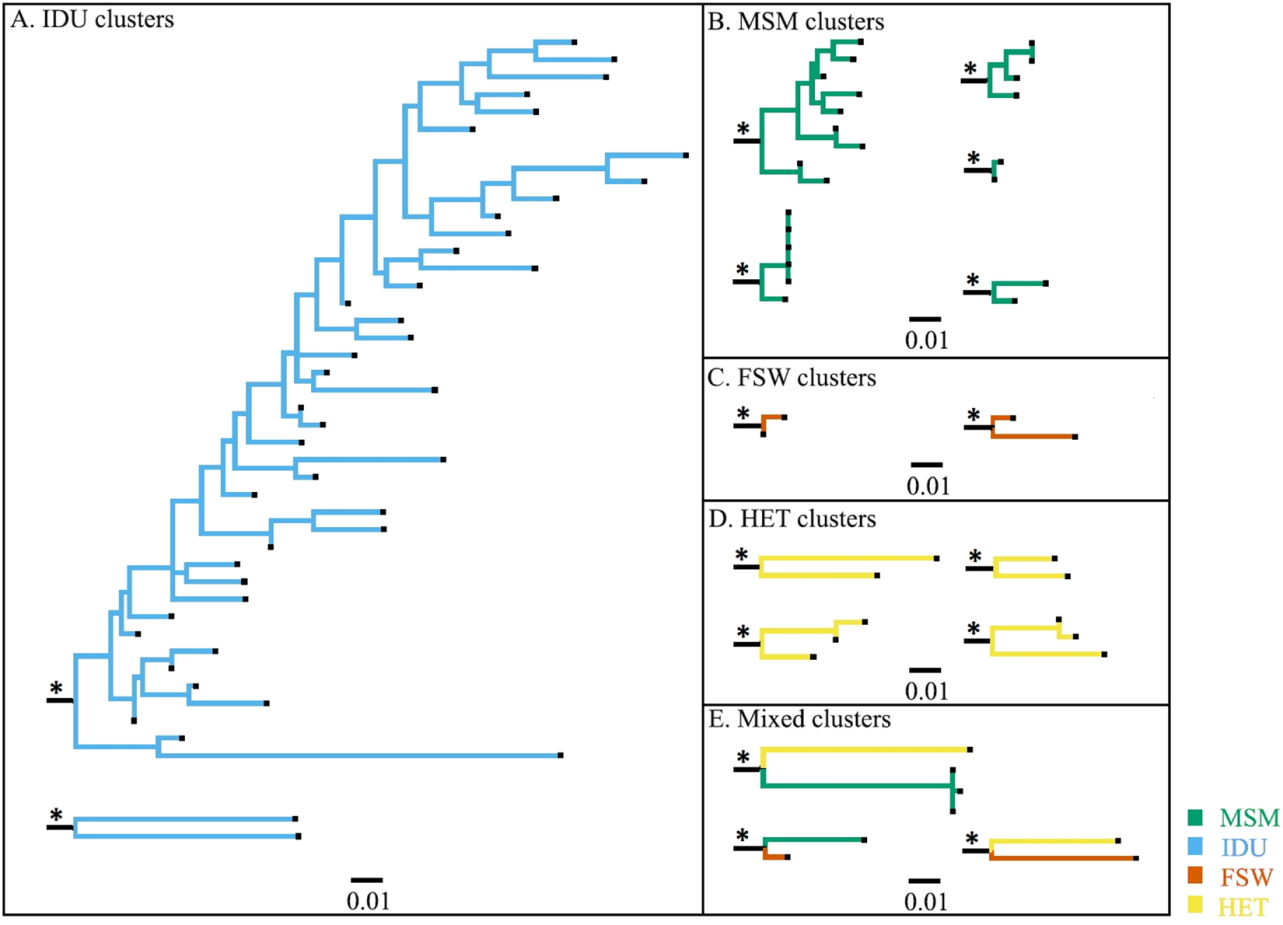
Clustering patterns of different risk groups in coastal Kenya. Representative clusters selected to highlight typical clustering patterns of the different risk groups. The branches are coloured according to the different risk groups (Bluish- green: MSM; Sky blue: IDU; Vermillion: FSW; Yellow: HET, and Black: reference sequences). As an overview, MSM formed several small clusters ranging in size from two to nine sequences per cluster (**A**). Most IDU sequences (N = 41) formed one single large cluster (**B**). In contrast, FSW and HET clusters were small (mostly dyads containing two sequences), although most FSW and HET sequences existed as single sequences or clustered with reference sequences (**C**). Asterisks have been used to highlight branches leading to significantly supported clusters (aLRT-SH branch support of ≥0.9).

Stratification by risk group showed two distinct clustering patterns (Fig. 1). The first pattern represented exclusive within-risk group clustering, where sequences in a cluster belonged exclusively to one specific risk group. Compared to HET sequences, MSM and IDU sequences were more likely to cluster (adjusted odds ratio [aOR] 25.9, 95% confidence interval [CI] 10–63.9, P < 0.001; and aOR 31.5, CI 12.2–81.6, P < 0.001, respectively, Table 3). Of the 61 clusters observed, 85% were risk group exclusive clusters. These included 24 MSM clusters (11 dyads and 13 networks), four IDU clusters (three dyads and one large cluster), six FSW clusters (six dyads), and 18 HET clusters (13 dyads and five networks). The majority of the MSM sequences (N = 84, 64%) formed small independent clusters ranging in size from two to nine sequences per cluster. Likewise, the majority of IDU sequences (N = 47, 82%) formed clusters. Interestingly, most of the clustering IDU sequences were of sub-subtype A1 and formed one single large cluster (N = 41, 80%). In contrast, only a small proportion of the FSW (N = 22, 20%) and HET (N = 67, 18%) sequences formed risk group-exclusive clusters.

**Table 3:** Factors associated with clustering among 546 subtypes A1, C, and D HIV-1 sequences from MSM, IDU, FSW, and HET individuals from coastal Kenya.

Characteristics		Bivariable Analysis^*^		Multivariable Analysis^**^	
		OR (95% CI)	P*-*value	aOR (95% CI)	P-value
**Risk group**	HET	Reference		Reference	
	MSM	13.8 (8.6–22.2)	<0.001	25.8 (10–63.9)	<0.001
	IDU	29.1 (13.8–61.1)	<0.001	31.5 (12.2–81.6)	<0.001
	FSW	1.1 (0.6–2)	0.71	1.2 (0.5–2.9)	0.656
**Subtype**	A1	Reference			
	C	0.4 (0.2–0.8)	0.01	0.4 (0. 1–0.9)	0.025
	D	0.5 (0.3–0.8)	0.008	0.4 (0.2–0.8)	0.014
**Year (range)**	2005–2007	Reference			
	2008–2010	0.9 (0.6–1.4)	0.55		
	2011–2019	1.1 (0.6–1.9)	0.83		
**Area (county)**	Kilifi	Reference			
	Mombasa	3.9 (2.7–5.5)	<0.001	0.9 (0.5–1.6)	0.675
**Sequence category**	Published	Reference			
	New	0.3 (0.2–0.5)	<0.001	0.8 (0.4–1.8)	0.555

Abbreviations: MSM, men who have sex with men; IDU, injecting drug user; FSW, female sex worker; HET, at-risk men and women who did not report sex work or male same-sex behaviour.

^*^Variables at a P-value of <0.1 in the bivariable analysis were included in the multivariable model.

Circulating and unique recombinant forms were excluded from the multivariable analysis.

In addition to risk group-exclusive clustering, 15% of all clusters were mixed between risk-groups (Table 2): Two (3%) MSM/FSW dyads, four (7%) MSM/HET mixed clusters (two dyads and two networks), and three (5%) FSW/HET clusters (two dyads and one network). Of relevance, both MSM/HET networks (one sub-subtype A1 and one subtype D) had sequences from four MSM and one HET female. Moreover, of the eleven MSM sequences that were found in mixed clusters, eight (73%) reported sex work in the three months preceding sample collection, and 10 (91%) reported bisexual behaviour. The FSW/HET network consisted of two FSW sequences and one HET male sequence.

Most sequences from coastal Kenya clustered exclusively with sequences of Kenyan origin. Only three clusters with sequences from coastal Kenya had links to published sequences from outside coastal Kenya. One sequence in an MSM cluster was from an MSM from Nairobi. One sequence in another MSM cluster was from a German MSM, and the last sequence was found in a mixed MSM/HET cluster and was from a Canadian individual of unknown gender (Supplementary Table S3).

### Genetic diversity between clusters of different risk groups

**Figure 2:**
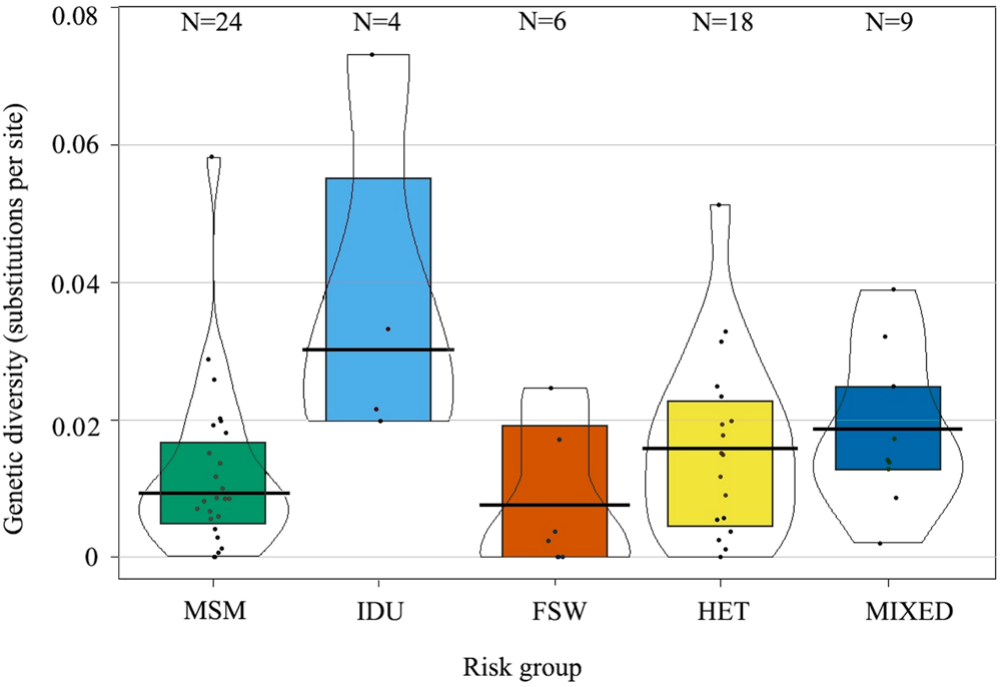
Genetic diversity of different risk group-specific clusters in coastal Kenya. A pirate plot^63^ illustrating the differences in genetic diversity between MSM, IDU, FSW, HET and Mixed clusters. Black dots represent the median estimates of the genetic diversity per cluster. The group median and the interquartile range diversity estimates are indicated in box plots coloured by risk group (Bluish-green: MSM; Sky blue: IDU; Vermillion: FSW; Yellow: HET; Deep blue: Mixed risk groups).

To further dissect differences in clustering patterns between risk groups, we determined the average genetic diversity in the identified clusters. A previously described ML bootstrap approach was employed to determine the genetic diversity (based on 1000 ML bootstrap trees)^37^. The median genetic diversity was 0.009 substitutions/site (s/s, IQR: 0.005–0.017 s/s) for MSM clusters, 0.03 s/s (IQR: 0.02–0.055 s/s) for IDU clusters, 0.008 s/s (IQR: 1×10^–8^–0.018 s/s) for FSW clusters, 0.015 s/s (IQR: 0.006–0.023 s/s) for HET clusters, and 0.018 s/s (IQR: 0.013–0.024 s/s) for mixed clusters. A Kruskal-Wallis H test showed that the distribution of genetic diversity differed across the five groups (χ^2^ = 11.074, four degrees of freedom, P-value = 0.026). A Dunn’s post hoc test for multiple comparisons using rank sums showed a significant difference in diversity between FSW and IDU (mean rank difference = 33.08, adjusted P-value = 0.039, Fig. 2, Supplementary Table S4).

### Analysis of active transmission clusters

Among the 61 clusters defined by risk group, we identified 34 potentially active clusters as (determined by low genetic distance <1.5%), suggesting ongoing transmission at the time of sample collection. Stratification of the potentially active clusters by risk-group showed: Eight MSM dyads and five MSM networks; four IDU dyads; five FSW dyads; and seven HET dyads and two HET networks. Potentially active clusters with sequences from different risk groups included one FSW/HET network and two MSM/HET networks (Supplementary Table S5).

### Estimation of time to the most recent common ancestor (tMRCA) and evolutionary rates

**Figure 3:**
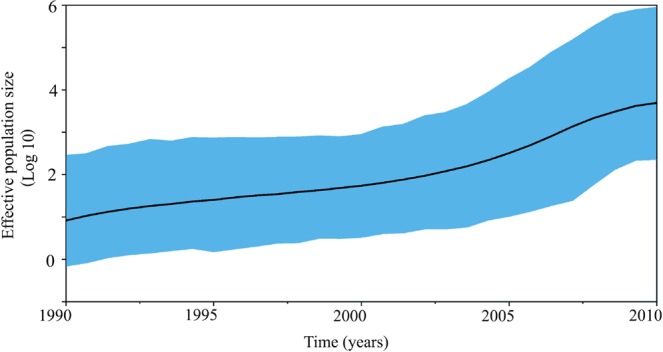
Population dynamics of the HIV-1 sub-epidemic among injecting drug users in coastal Kenya. A Bayesian Skygrid plot showing population dynamics of the HIV-1 sub-subtype A1 injecting drug users’ sub-epidemic in coastal Kenya. Since the IDU *pol* sequence alignment did not contain temporal information (all sequences were sampled in 2010), the node height for this cluster was calibrated using information from the tMRCA posterior distribution obtained from dating the origin of subtype A1 Kenyan clusters^64^. Median estimates of the number of injecting drug users contributing to new infections are shown as a continuous black line. The shaded area represents the 95% higher posterior density intervals of the inferred effective population size.

To gain insight into the evolutionary dynamics of the identified transmission clusters, we determined the evolutionary rate and the tMRCA of the clusters by Bayesian phylogenetic analysis. The median tMRCA of the 61 coastal Kenya clusters indicated that HIV-1 has been introduced in coastal Kenya several times over a period of 27 years (1985–2012, Supplementary Fig. S3 and Table S3). Only one cluster was large enough to allow for in-depth phylodynamic analysis. This cluster comprised 41 IDU sequences and the tMRCA for this cluster was determined to be 1987 (95% higher posterior density [HPD] interval: 1985–1990) (Fig. 3) with a median evolutionary rate of 6.4 × 10^−3^ substitutions/site/year (HPD interval: 3.9 × 10^−3^ − 1.1 × 10^−2^). The Skygrid analysis indicated that the number of IDU contributing to new HIV-1 infections over time increased gradually from 1987 to 2010.

## Discussion

In this study, we found several HIV-1 links between MSM/HET, HET/FSW, and MSM/FSW, indicating mixing between these risk groups in coastal Kenya. Sequences from HET females in clusters dominated by MSM sequences provided evidence for heterosexual linkages in these clusters. The majority of the MSM in mixed clusters also reported having female sexual partners, indicating that this group, in addition to female sex workers, could be an important transmission link to the HET epidemic^4^.

Transmission linkages between MSM and HET in coastal Kenya might be expected to some extent, given that sexual interaction between MSM and other risk groups in the community is common^2–4,6,13^. In the only previous study of phylogenetic HIV-1 transmission linkages between MSM and HET in coastal Kenya, Bezemer and colleagues only found one single transmission pair of an MSM and a known female partner. Hence, extensive mixing between the MSM and HET epidemics was not found in that study^6^. In comparison, our analysis included a higher sampling density and availability of risk-group annotated sequences obtained in more recent years. This likely explains the significantly higher number of mixed clusters between MSM and HET sequences in the current study. In a broader context, our study complements existing research on the role of mixed networks in HIV-1 transmission – both globally and in sub-Saharan Africa^26,30,31,38–40^.

Although we found several instances of mixed clusters, the majority of the coastal Kenya clusters represented within-risk-group HIV-1 transmission. MSM-exclusive and IDU-exclusive clusters were more common than FSW and HET clusters. High rates of clustering among MSM and IDU have been described before, both in our setting and elsewhere, and have been linked to an elevated risk of infection among MSM and IDU within close networks^6,8,17,29,30,41^. Whereas the MSM sequences were found in several smaller clusters, the vast majority of the IDU sequences formed one large cluster. This suggests that the majority of the HIV-1 IDU epidemic in Coastal Kenya was introduced from one single source followed by a long-term gradual spread within the IDU population – a pattern that distinguishes IDU transmission from that of other risk groups in coastal Kenya. In contrast to previous studies where IDU sequences clustered with very low genetic diversity^29,30,42^, IDU clusters in our analysis had the highest genetic diversity compared to the other analysed risk groups. This indicates that the elapsed time between HIV-1 infection and sample collection may have been longer among IDUs, and/or that the proportion of collected IDU sequences in relation to the true number of IDUs was lower compared to other risk groups. The underlying reasons for this are unknown and warrant further investigation. However, with or without missing links, such clustering pattern is indicative of long-standing HIV-1 transmission linked with intravenous drug use in coastal Kenya^17^.

This is the first study in Africa to investigate the evolutionary dynamics of an HIV-1 sub-epidemic among IDU. The phylodynamic analysis indicated a steady increase in the epidemic among IDU in coastal Kenya from 1987 to 2010, with no evidence of a rapid exponential increase in the number infections that is typical of HIV-1 outbreaks among IDUs^29,30,42^. Still, the gradual increase in infections among IDU is compatible with a known period of increased injection of heroin in the region^43^. Interestingly, and given a general absence of epidemiological surveillance data, new infections among IDU did not seem to decrease with the national rollout of combination antiretroviral therapy (ART) in 2004. This could be a consequence of the unfavourable climate of stigma, discrimination and hostile legislation associated with IDU and most-at-risk-populations in Kenya, which impedes these populations from accessing medical services including ART^1,44^. Opioid substitution therapy for IDU, Pre-Exposure Prophylaxis (PrEP) targeting all high-risk groups, and initiation of ART immediately upon diagnosis have all been introduced and scaled up after 2010, when the IDU samples used for this study were collected^45,46^. As new sequence data are made available, future studies may shed light on the effectiveness of these strategies.

This study had some limitations: First, the identified transmission chains are likely to suffer from missing links due to low sampling density. A low sampling density generally results in reduced clustering of HIV-1 sequences^47^. Because majority of the studies in the coastal Kenya setting have mostly focused on recruiting MSM participants, FSW and HET in our analysis had low sampling densities and inevitably, several transmission chains may have been missed. Furthermore, it is important to acknowledge that the IDU sequences were from one study, using samples from one setting (Mombasa), and collected over a period of less than one year (2010). It is therefore likely that our findings are not representative of the entire IDU epidemic in coastal Kenya. Larger studies of HIV-1 transmission in the IDU population in coastal Kenya are therefore warranted; still we found clear branching patterns indicative of long-standing HIV-1 transmission associated with intravenous drug use. Second, skewed sampling between risk groups may result in overrepresentation of some types of risk group-specific and mixed clusters. Third, given that annotating sequences from sub-Saharan Africa with information about transmission risk factor has become common only in recent years, some published sequences used in this analysis lacked risk data and were assigned HET (by far the most dominant route of HIV-1 transmission in coastal Kenya). However, the risk group for nodes within a cluster that had inadequate annotation can often be deduced from association with nodes with a known risk group^48^. Since none of the presumed heterosexual sequences in this study was found in mixed clusters, it is unlikely that this potential caveat had any effect on our conclusions. Finally, few HIV-1 *pol* sequences were available after the year 2010 for all risk groups. This limited our analysis to the representation of some risk groups by the year and area of sampling, further restraining characterisation of recent clusters and ongoing transmission chains.

In conclusion, we demonstrated that there is HIV-1 mixing between high-risk groups and heterosexual populations in coastal Kenya, with frequent within-risk-group transmission. We highlight that high-risk groups could contribute to the epidemic either through seeding and maintaining new infections within their own risk group or through linking infections across different risk groups. It is possible that HIV-1 prevention programmes targeting FSW, MSM and IDU populations could reduce overall HIV-1 transmission in coastal Kenya. As more sequences become available from wider geographic regions, further and larger studies with uniform sampling densities across different risk groups will be necessary to estimate the impact of mixing between risk groups and the general population on HIV-1 spread and to determine the source populations that could most effectively be targeted to mitigate new infections in sub-Saharan Africa.

## Methods

### Study population and sequence dataset

All published HIV-1 *pol* sequences available in the HIV database at the Los Alamos National Laboratory (LANL) originating from coastal Kenya and collected 2005–2019 retrieved^49^. Sequences sampled from the same individuals were excluded from the data set, retaining only the oldest sequence per participant. Risk group information was obtained from LANL and any missing data were obtained by communication with study authors or inferred from reviewing literature from the respective studies^6,8–15^. In addition, new HIV-1 *pol* sequences were generated from samples collected 2005–2019 participants in an acute HIV-1 infection cohort and from a prospective observational study following high-risk volunteers in an HIV-1 vaccine feasibility cohort described elsewhere^3^. All new sequences were collected from treatment-naïve men and women aged 18 years and over. HIV-1 diagnosis for samples collected before 2016 was done using two rapid antibody tests in parallel (Determine, Abbott Laboratories; Unigold, Trinity Biotech), with conflicting results resolved by an enzyme-linked immunosorbent assay (ELISA, Genetic System HIV-1/2 plus O EIA; Bio-Rad). HIV-1 diagnosis for samples collected after 2016 was made using the GeneXpert HIV-1 Qual (Cepheid, Sunnyvale, CA, USA).

Sequences were annotated by date of sample collection, geographical area (Mombasa or Kilifi county), and by risk group. Sequences were classified into: MSM (men who reported having sex with men); IDU (individuals who reported injecting drugs with a needle and syringe); FSW (females who reported ever receiving money, gifts, or favours in exchange for sex); and HET (all other individuals [both male and female] who did not report engaging in sex work, male same-sex behaviour or injection drug use).

### RNA extraction, amplification of HIV-1 pol region and sequencing

HIV-1 RNA was extracted from blood plasma samples using the RNeasy Lipid Tissue Mini Kit (QIAGEN) with modifications from the manufacturer’s standard protocol^50^. Briefly, 100 µl of blood plasma was efficiently lysed in 1000 µl Qiazol Lysis Reagent (Qiagen). DNA was removed by treating the column with RNase-free DNase 1 (Qiagen) prior to RNA elution in 40 µl nuclease-free water. Reverse transcription and amplification of partial *pol* gene were performed using the One-Step Superscript III RT/Platinum Taq High Fidelity Enzyme Mix (ThermoFisher Scientific^TM^) with the *pol*-specific primer pair JA269 and JA272^51^. First-round PCR products were amplified in a nested PCR with DreamTaq Green DNA Polymerase (ThermoFisher Scientific^TM^) using *pol-*specific primers JA271 and JA270. PCR products were sequenced in both directions with the nested PCR primers using the BigDye terminator kit v1.1 (Applied Biosystems) and the sequences were determined on an ABI PRISM 3130×1 Genetic Analyzer (Applied Biosystems).

### Sampling density

County-exclusive estimates for HIV-1 prevalence for high-risk groups in Kenya were not available when this analysis was done. Hence, the national HIV-1 prevalence estimate for each risk group and the estimates of people infected with HIV-1 in Mombasa and Kilifi counties were used to estimate the sampling density of our study (defined as the proportion of genotyped viral sequences in the estimated number of HIV-infected individuals in a risk group)^36^.

### HIV-1 Subtyping

Newly generated consensus and published *pol* sequences from coastal Kenya were combined, codon-aligned using ClustalX 2.0.11, and manually adjusted in Geneious Prime 2019 (Version 2019 2.1) (https://www.geneious.com)^52^. The combined sequences were then aligned with the Group M (subtypes A-K + Recombinants) HIV-1 subtype reference dataset downloaded from Los Alamos HIV Database (http://www.hiv.lanl.gov/)^49^.

To infer genetic relatedness, phylogenetic reconstruction was done in PhyML using the general time-reversible substitution model with a gamma-distributed rate variation and proportion of invariant sites (GTR + Γ4 + Ι)^53^. Branch support was estimated using the Shimodaira-Hasegawa approximate likelihood ratio test (aLRT-SH) as implemented in PhyML^54^. An aLRT-SH ≥0.9 was considered statistically significant^17^. The subtype-resolved maximum-likelihood phylogenetic tree was visualized in FigTree (v1.4.3). Unique recombinant forms (URFs) were further resolved by Bootscan analyses using SimPlot and breakpoints identified using a sliding window size of 300 bp and a step size of 20 bp^55^.

### Transmission Cluster analysis

To detect local transmission clusters, newly generated and published HIV-1 *pol* sequences were combined into one coastal Kenya sequence dataset. In addition, the ten most similar GenBank reference sequences for each coastal Kenya sequence were obtained through BLAST^29,30,56^. The unique coastal Kenya sequences and the reference sequences were aligned and analysed to determine HIV-1 transmission clusters. Subtype-specific maximum-likelihood phylogenies were reconstructed in PhyML. For each subtype, transmission clusters were manually determined by inspecting the ML tree from root to terminal tips to ensure sequences clustered with high branch support. Monophyletic clades with aLRT-SH support ≥0.9 and which were dominated (≥80%) by sequences from coastal Kenya (compared to reference sequences) were defined as coastal Kenya transmission clusters^17^. To determine active transmission clusters, sequences were further explored using a genetic distance threshold (≤1.5%) and aLRT-SH branch support of ≥0.9 in Cluster Picker^57^. Transmission networks were classified based on the number of sequences per cluster into dyads (2 sequences), networks (3–14 sequences) and large clusters (>14 sequences)^30^.

### Diversity analysis

A previously described ML bootstrap approach was employed to determine the genetic diversity in the identified clusters^37^. Briefly, all sequences in coastal Kenya transmission clusters were used to construct 1000 bootstrap ML phylogenies in Garli 2.0^58^. Diversity estimates were determined in Perl (version 5.30.0) using in-house Perl and Bioperl scripts^59^. The diversity for each pre-defined cluster was estimated by averaging the pairwise tree-distances between the cluster-specific sequences in each bootstrap tree, resulting in 1000 diversity estimates per cluster. Next, the medians of these 1000 diversity estimates were determined for each cluster and then used in the analysis as previously described^37^. The scripts used in this analysis is available from the authors upon request.

### Bayesian phylogenetic analysis

To estimate the dates of origin (time to most recent common ancestor; tMRCA) of the coastal Kenyan clusters, maximum clade credibility trees were generated using a Bayesian Markov Chain Monte Carlo (MCMC) approach as implemented in BEASTv1.8.2^60,61^. Based on marginal likelihood estimators for model selection and testing in BEAST, the Bayesian Skygrid model with an uncorrelated lognormal relaxed clock and inferred under the GTR + Γ4 + Ι substitution model was adopted as the best fit model for subsequent inferences. BEAST runs of 100–300 million generations were performed, logging samples after every 10000–30000 steps with the initial 10–30% discarded as burn-in. The convergence of MCMC parameter estimates was accessed based on effective sample size estimates (ESS > 200) using Tracer v1.6^62^. Trees were summarized in Tree-Annotator v1.8.2 (BEAST suite) and maximum clade credibility (MCC) trees were visualized in Figtree. We also aimed to dissect the demographic history of the only large coastal Kenya cluster identified. Since the sequences in this cluster did not contain temporal information (all IDU sequences were sampled in 2010), the node height posterior distribution (tMRCA) for the IDU cluster from the transmission clusters analysis described above was used as a tree-root height calibration prior (fixed the tree root height to 1985), guiding a skygrid analysis to estimate the effective population size (*N*_*e*_) of the large IDU cluster over time.

### Statistical analysis

Frequencies and percentages were used to describe the distribution of sequences within the study population by risk group, HIV-1 subtype, calendar year of sampling and county of sampling. A logistic regression model was used to assess factors associated with clustering. Variables with P-values <0.1 in the bivariable analysis were included in the multivariable model. A P-value of <0.05 was defined as statistically significant. A Kruskal-Wallis H test and a post hoc Dunn’s test (with Bonferroni correction for multiple comparisons) were conducted to determine differences in genetic diversity estimates among clusters from multiple risk groups. Statistics were done using Stata version 15 and RStudio (version 1.2.5001), and the packages: *DescTools* (version 0.99.29, https://cran.r-project.org/package=DescTools) and *yarrr* (version 0.1.6)^63^. The full R code is available on request from the authors.

### Nucleotide sequence accession numbers

Nucleotide sequences were deposited in GenBank under the following accession numbers: MT084914 - MT085076.

### Ethical consideration

All research was performed in accordance with relevant guidelines/regulations. Informed consent was obtained from all participants who provided blood plasma samples from which new HIV-1 sequences were generated (informed consent was not required for subjects whose sequences were obtained from LANL). Plasma samples used to generate the new sequences were obtained from on-going or concluded studies that were also approved by KEMRI/SERU (SERU 3747, 3280 and 3520, and SSC 894). The current study is part of a parent protocol that was reviewed and approved by the Kenya Medical Research Institute (KEMRI) Scientific and Ethics Review Unit (SERU 3547).

## Supplementary information

Supplementary information.
