# Supplementary material for: HIV-1 Transmission Patterns Within and Between Risk Groups in Coastal Kenya
Source: Sci Rep. 2020 Apr 21;10:6775. doi: 10.1038/s41598-020-63731-z (PMC7174422; doi:10.1038/s41598-020-63731-z)
Supplement: Supplementary file 1 — Supplementary information. [file 41598_2020_63731_MOESM1_ESM.docx]

**HIV-1 TRANSMISSION PATTERNS WITHIN AND BETWEEN RISK GROUPS IN COASTAL KENYA**

**George M. Nduva^1,2^, Amin S. Hassan^1,2^, Jamirah Nazziwa^1^, Susan M. Graham^2,3^, Joakim Esbjörnsson^1,4*^, and Eduard J. Sanders^2,4*^**

**^1^**Lund University, Lund, Sweden, **^2^**KEMRI/Wellcome Trust Research Programme, Kilifi, Kenya, **^3^**University of Washington, Seattle, WA, USA,  **^4^**The University of Oxford, Oxford, United Kingdom.

^*^These authors contributed equally to this work.

**Corresponding Author:**

Joakim Esbjörnsson

BMC B13

Department of Translational Medicine

Lund University

221 84 Lund, Sweden

Email: [Joakim.esbjornsson@med.lu.se](mailto:Joakim.esbjornson@med.lu.se)

**Files in this Data Supplement:**

Table S1. The proportions of genotyped viral sequences in the estimated number of HIV-infected MSM, IDU, FSW and HET individuals from coastal Kenya.

Table S2. Sim-Plot analysis results for unique and circulating recombinants forms among newly generated sequences and their respective recombination breakpoints.

Table S3. Characteristics and posterior distribution of time to most recent common ancestors estimated for coastal Kenya transmission clusters.

Table S4. A summary of Dunn’s post hoc test of multiple comparisons using rank sums indicating the differences in diversity between coastal Kenyan clusters.

Table S5. Number of coastal Kenyan active transmission clusters at a genetic distance threshold of <1.5%.

Legends for supplementary figures.

Figure S1. Maximum-likelihood tree used to determine the HIV-1 subtype of the analysed sequences.

## Figure S2. Maximum-likelihood trees used to identify transmission clusters.

Figure S3. Maximum clade credibility trees used to date clusters.

# **TABLES**

**Table S1. The proportions of genotyped viral sequences in the estimated number of HIV-infected MSM, IDU, FSW and HET individuals from coastal Kenya.**

| **Risk group** | **Risk group population estimates*** | **Population infected with HIV-1**** | **Study sample size (N)^***^** | **Sampling density (%)**^#^ |
| --- | --- | --- | --- | --- |
| **MSM** | 1,422 | 256 | 131 | 51% |
| **IDU** | 2,610 | 470 | 58 | 12% |
| **FSW** | 13,964 | 4050 | 109 | 3% |
| **HET** | 1,491,423 | 73080 | 360 | 0.5% |

MSM, men who have sex with men; IDU, injecting drug user; FSW, female sex worker; HET, at-risk men and women who did not report sex work or male same-sex behaviour.

^*^Estimated number of adult MSM, IDU, FSW and HET in Mombasa and Kilifi counties^1^.

^**^Estimated population infected with HIV-1 in Mombasa and Kilifi counties^1,2^.

^***^Number of HIV-1 infected individuals included in the study based on estimated population infected with HIV-1 in Mombasa and Kilifi .

^#^Proportions of HIV-1 infected individuals included in the study.

## **Table S2. Sim-Plot analysis results for unique and circulating recombinants forms among newly generated sequences and their respective recombination breakpoints.**

| **Risk group** | **Patient** | **Recombinant^**^** | **Subtype** | **Breakpoint** | **Subtype** | **Breakpoint** | **Subtype** |
| --- | --- | --- | --- | --- | --- | --- | --- |
| FSW | 1511 | A1_16A2D | A1 | 655 | 16.A2D |  |  |
| HET | 1027 | A1_A2_16A2D | A1 | 518 | A2 | 638 | 16.A2D |
| FSW | 506 | A1_D | A1 | 685 | D |  |  |
| HET | 957 | A1_D | A1 | 675 | D |  |  |
| HET | 1105 | A1_D | D | 329 | A1 | 640 | D |
| FSW | 1736 | A1_D | A1 | 584 | D |  |  |
| FSW | 1789 | A1_D | A1 | 224 | D |  |  |
| FSW | 1845 | A1_D | A1 | 303 | D |  |  |
| FSW | 1616 | A2_16A2D | A2 | 278 | 16A2D | 633 | A2 |
| HET | 4304 | A2_16A2D | A2 | 280 | 16A2D | 625 | A2 |
| FSW | 1816 | A2_16A2D | A2 | 283 | 16_A2D | 443 | A2 |
| FSW | 1894 | A2_16A2D | A2 | 195 | 16A2D | 485 | A2 |
| HET | 793 | A2_16A2D | 16A2D | 486 | A2 | 637 | 16A2D |
| FSW | 1755 | A2_16A2D | 16_A2D | 696 | A2 |  |  |
| FSW | 1085 | C_A1 | A1 | 165 | C |  |  |
| FSW | 1780 | C_A1 | A1 | 198 | C |  |  |
| FSW | 1953 | C_A1 | A1 | 195 | C |  |  |
| HET | 8971 | C_A1 | C | 285 | A1 | 743 | C |
| FSW | 1918 | C_A1_D | A1 | 183 | C | 691 | D |
| MSM | 2760 | A1_D | A1 | 251 | D |  |  |
| MSM | 2643 | A1_D | A1 | 257 | D |  |  |
| MSM | 2913 | A1_D | A1 | 255 | D |  |  |
| MSM | 2801 | A1_D | A1 | 255 | D |  |  |
| MSM | 2787 | A1_D | A1 | 255 | D |  |  |
| MSM | 2891 | A1_C | A1 | 261 | C |  |  |

MSM, men who have sex with men; IDU, injecting drug user; FSW, female sex worker; HET, at-risk men and women who did not report sex work or male same-sex behaviour.

^**^Circulating recombinant forms and unique recombinant forms.

**Table S3. Characteristics and posterior distribution of time to most recent common ancestors estimated for coastal Kenya transmission clusters.**

| **Cluster name^1^** | **Sequences** | **Risk group^2^** | | | | **Year(s) of diagnosis** | **tMRCA^3^** |
| --- | --- | --- | --- | --- | --- | --- | --- |
|  | **N** | **MSM** | **IDU** | **FSW** | **HET** |  |  |
| A1_FSW_1 | 2 | - | 0 | 2 | 0 | 2007 | 1996 |
| A1_FSW_2 | 2 | - | 0 | 2 | 0 | 2005-2008 | - |
| A1_FSW_3 | 2 | - | 0 | 2 | 0 | 2006-2008 | 2005 |
| A1_FSW_4 | 2 | - | 0 | 2 | 0 | 2006 | 2006 |
| A1_FSW_5 | 2 | - | 0 | 2 | 0 | 2006 | 2006 |
| A1_FSW_6 | 2 | - | 0 | 2 | 0 | 2006 | 2005 |
| A1_HET_1 | 2 | 0 | 0 | 0 | 2 | 2008-2009 | 1999 |
| A1_HET_2 | 2 | 0 | 0 | 0 | 2 | 2008-2010 | 2005 |
| A1_HET_3 | 2 | 0 | 0 | 0 | 2 | 2008 | 2004 |
| A1_HET_4 | 2 | 0 | 0 | 0 | 2 | 2009 | 2009 |
| A1_HET_5 | 2 | 0 | 0 | 0 | 2 | 2009-2016 | 1990 |
| A1_HET_6 | 2 | 0 | 0 | 0 | 2 | 2009-2010 | 2003 |
| A1_HET_7 | 2 | 0 | 0 | 0 | 2 | 2008 | 1999 |
| A1_HET_8 | 2 | 0 | 0 | 0 | 2 | 2009 | 2005 |
| A1_HET_9 | 2 | 0 | 0 | 0 | 2 | 2008 | 2007 |
| A1_HET_10 | 3 | 0 | 0 | 0 | 3 | 2008-2009 | 1997 |
| A1_HET_11 | 3 | 0 | 0 | 0 | 3 | 2008 | 1998 |
| A1_HET_12 | 3 | 0 | 0 | 0 | 3 | 2008-2016 | 1998 |
| A1_HET_13 | 4 | 0 | 0 | 0 | 4 | 2008-2016 | 1998 |
| A1_HET_14 | 4 | 0 | 0 | 0 | 4 | 2008-2009 | 2001 |
| A1_IDU_1 | 2 | 0 | 2 | 0 | 0 | 2010 | 1985 |
| A1_IDU_2 | 41 | 0 | 41 | 0 | 0 | 2010 | 1985 |
| A1_Mixed_1 | 2 | 0 | 0 | 1 | 1 | 2006-2008 | 2003 |
| A1_Mixed_2 | 2 | 1 | 0 | 1 | 0 | 2009-2012 | 2003 |
| A1_Mixed_3 | 2 | 0 | 0 | 1 | 1 | 2009-2017 | - |
| A1_Mixed_4 | 2 | 1 | 0 | 1 | 0 | 2005-2017 | 2002 |
| A1_Mixed_5 | 2 | 1 | 0 | 0 | 1 | 2006-2008 | 1996 |
| A1_Mixed_6 | 2 | 1 | 0 | 1 | 0 | 2006-2017 | 1994 |
| A1_Mixed_7 | 3 | 0 | 0 | 1 | 2 | 2014 | 2012 |
| *A1_Mixed_8 | 5 | 4 | 0 | 0 | 1 | 2006-2017 | 1995 |
| A1_MSM_1 | 2 | 2 | 0 | - | 0 | 2015-2018 | 2010 |
| A1_MSM_2 | 2 | 2 | 0 | - | 0 | 2009 | 2009 |
| A1_MSM_3 | 2 | 2 | 0 | - | 0 | 2010 | 2010 |
| A1_MSM_4 | 2 | 2 | 0 | - | 0 | 2014 | 2008 |
| A1_MSM_5 | 2 | 2 | 0 | - | 0 | 2016 | 2005 |
| A1_MSM_6 | 2 | 2 | 0 | - | 0 | 2009 | 2008 |
| A1_MSM_7 | 2 | 2 | 0 | - | 0 | 2006 | 2005 |
| A1_MSM_8 | 2 | 2 | 0 | - | 0 | 2009 | 2004 |
| A1_MSM_9 | 2 | 2 | 0 | - | 0 | 2009-2011 | 1997 |
| A1_MSM_10 | 3 | 3 | 0 | - | 0 | 2008-2009 | 2005 |
| A1_MSM_11 | 3 | 3 | 0 | - | 0 | 2008-2009 | 2002 |
| A1_MSM_12 | 3 | 3 | 0 | - | 0 | 2009-2016 | - |
| A1_MSM_13 | 3 | 3 | 0 | - | 0 | 2010-2014 | 2008 |
| A1_MSM_14 | 5 | 4 | 0 | - | 0 | 2006-2012 | 1998 |
| *A1_MSM_15 | 4 | 4 | 0 | - | 0 | 2006 | 2001 |
| A1_MSM_16 | 5 | 5 | 0 | - | 0 | 2007-2015 | 2001 |
| A1_MSM_17 | 5 | 5 | 0 | - | 0 | 2006-2019 | 2002 |
| *A1_MSM_18 | 6 | 6 | 0 | - | 0 | 2007-2012 | 1997 |
| A1_MSM_19 | 6 | 6 | 0 | - | 0 | 2009-2010 | 2005 |
| A1_MSM_20 | 9 | 9 | 0 | - | 0 | 2007-2013 | 1999 |
| C_HET_15 | 2 | 0 | 0 | 0 | 2 | 2008 | 2005 |
| C_IDU_3 | 2 | 0 | 2 | 0 | 0 | 2010 | 2000 |
| C_MSM_21 | 2 | 2 | 0 | - | 0 | 2008 | 1991 |
| C_MSM_22 | 4 | 4 | 0 | - | 0 | 2010-2016 | 2008 |
| D_HET_16 | 2 | 0 | 0 | 0 | 2 | 2007-2009 | 1986 |
| D_HET_17 | 2 | 0 | 0 | 0 | 2 | 2008-2009 | 1986 |
| D_HET_18 | 2 | 0 | 0 | 0 | 2 | 2007-2008 | 2007 |
| D_IDU_4 | 2 | 0 | 2 | 0 | 0 | 2010 | 1989 |
| D_Mixed_9 | 4 | 3 | 0 | 0 | 1 | 2006-2010 | 1986 |
| D_MSM_23 | 2 | 2 | 0 | - | 0 | 2009 | 2007 |
| D_MSM_24 | 3 | 3 | 0 | - | 0 | 2010-2011 | 2005 |

^1^Cluster named according to subtype/CRF, risk group dominating the cluster.

^2^MSM, men who have sex with men; IDU, injecting drug user; FSW, female sex worker; HET, at-risk men and women who did not report sex work or male same-sex behaviour.

^3^Median time to the most recent common ancestor of the cluster.

^*^Clusters containing sequences from outside coastal Kenya.

**Table S4. A summary of Dunn’s post hoc test of multiple comparisons using rank sums indicating the differences in diversity between coastal Kenyan clusters.**

| **Group** | **Mean rank difference** | **P-value (adjusted)** |
| --- | --- | --- |
| **HET-FSW** | 14.72 | 0.784 |
| **IDU-FSW** | 33.08 | 0.039* |
| **MIX-FSW** | 18.81 | 0.444 |
| **MSM-FSW** | 8.38 | 1 |
| **IDU-HET** | 18.36 | 0.612 |
| **MIX-HET** | 4.08 | 1 |
| **MSM-HET** | -6.35 | 1 |
| **MIX-IDU** | -14.28 | 1 |
| **MSM-IDU** | -24.71 | 0.099 |
| **MSM-MIX** | -10.43 | 1 |

MSM, men who have sex with men; IDU, injecting drug user; FSW, female sex worker; HET, at-risk men and women who did not report sex work or male same-sex behavior; MIX, mixed clusters having sequences belonging to different risk groups.

*Significant difference in genetic diversity between the two risk groups.

**Table S5. Number of coastal Kenyan active transmission clusters at a genetic distance threshold of <1.5%.**

| **Risk group** | **Number of active Transmission clusters (GD^#^ <1.5%)** | | |
| --- | --- | --- | --- |
|  | **Dyads (2 sequences)** | **Networks (3-14 sequences)** | **Total** |
| MSM | 8 | 5 | 13 |
| IDU | 4 | 0 | 4 |
| FSW | 5 | 0 | 5 |
| HET | 7 | 2 | 9 |
| FSW/HET | 0 | 1 | 1 |
| MSM/HET | 0 | 2 | 2 |

MSM, men who have sex with men; IDU, injecting drug user; FSW, female sex worker; HET, at-risk men and women who did not report sex work or male same-sex behaviour.

^#^Genetic distance between sequences in a cluster.

**FIGURE LEGENDS**

**Figure S1.** **Maximum-likelihood tree used to determine the HIV-1 subtype of the analysed sequences.**

Unrooted maximum-likelihood phylogenetic tree of 658 HIV-1 *pol* sequences from HIV-1 infected individuals from coastal Kenya. Branch tips colours correspond to the respective HIV-1 subtype, sub-subtype or recombinant form as shown in the legend. The tree is drawn to scale, with branch lengths measured in the number of substitutions per site. Key nodes with aLRT-SH support ≥0.9 are highlighted with an asterisk. Scale bars represent the genetic distance in substitutions per site in all phylogenies.

## **Figure S2. Maximum-likelihood trees used to identify transmission clusters**

Maximum-likelihood trees used for identification of coastal Kenya transmission clusters. Trees represent A: Sub-subtype A1; B: Subtype C; and C: Subtype D transmission clusters, respectively. Each phylogeny is rooted at the midpoint. Monophyletic clusters with aLRT-SH support ≥0.9 and which have ≥80% sequences from coastal Kenya are highlighted in grey. To enhance cluster visualization, some branches containing either reference sequences or coastal Kenya sequences that are not part of clusters have been collapsed (shown as black triangles, with the recent end of the triangle indicating the latest sampling date). Branch tips within respective clusters are coloured as per cluster risk group (Bluish- green: MSM; Sky blue: IDU; Vermillion: FSW; Yellow: HET; and Black: Reference sequences). Scale bars represent the genetic distance in substitutions per site in all phylogenies.

## **Figure S3. Maximum clade credibility trees used to date clusters**

Maximum clade credibility (MCC) trees used to determine the time to the most recent common ancestor of the coastal Kenya clusters. Trees represent A: Sub-subtype A1; B: Subtype C; and C: Subtype D, respectively. The node height for the sub-subtype A1 IDU cluster (dated to 1985) was used to calibrate a second analysis determining past population dynamics of the IDU cluster. Coastal Kenya clusters are highlighted in grey. To enhance cluster visualization, some branches containing either reference sequences or coastal Kenya sequences that are not part of clusters have been collapsed (shown as non-coloured triangles, with the recent end of the triangle indicating the latest sampling date. Branch tips are colour-coded as per risk group (Bluish- green: MSM; Sky blue: IDU; Vermillion: FSW; Yellow: HET; and Black: Reference sequences). Node bars represent 95% HPD estimates of the tMRCA to each cluster. Scale bars represent the genetic distance in substitutions per site in all phylogenies.

**FIGURES**

## **Figure S1.**

**
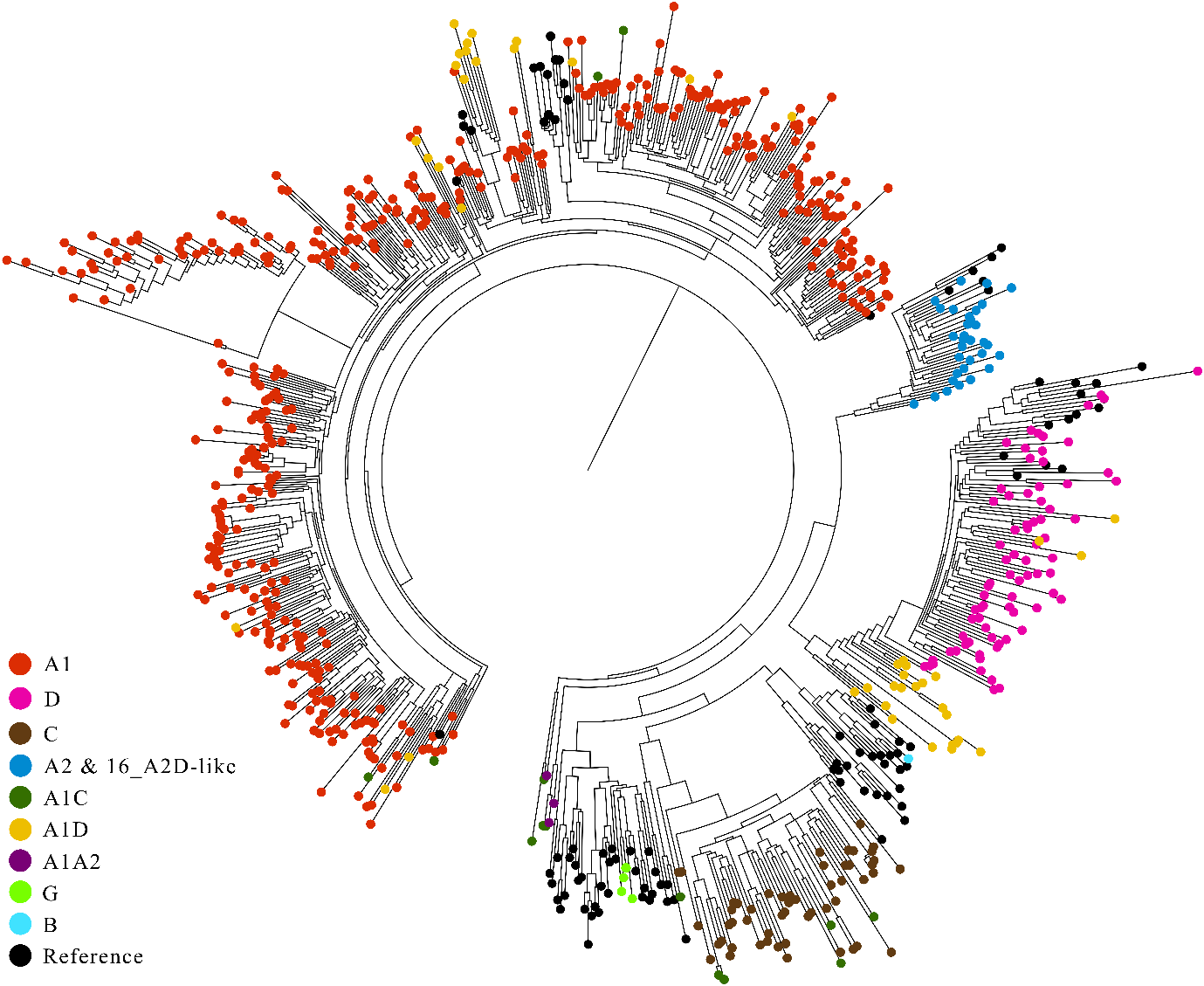
**

## **Figure S2.**

1.
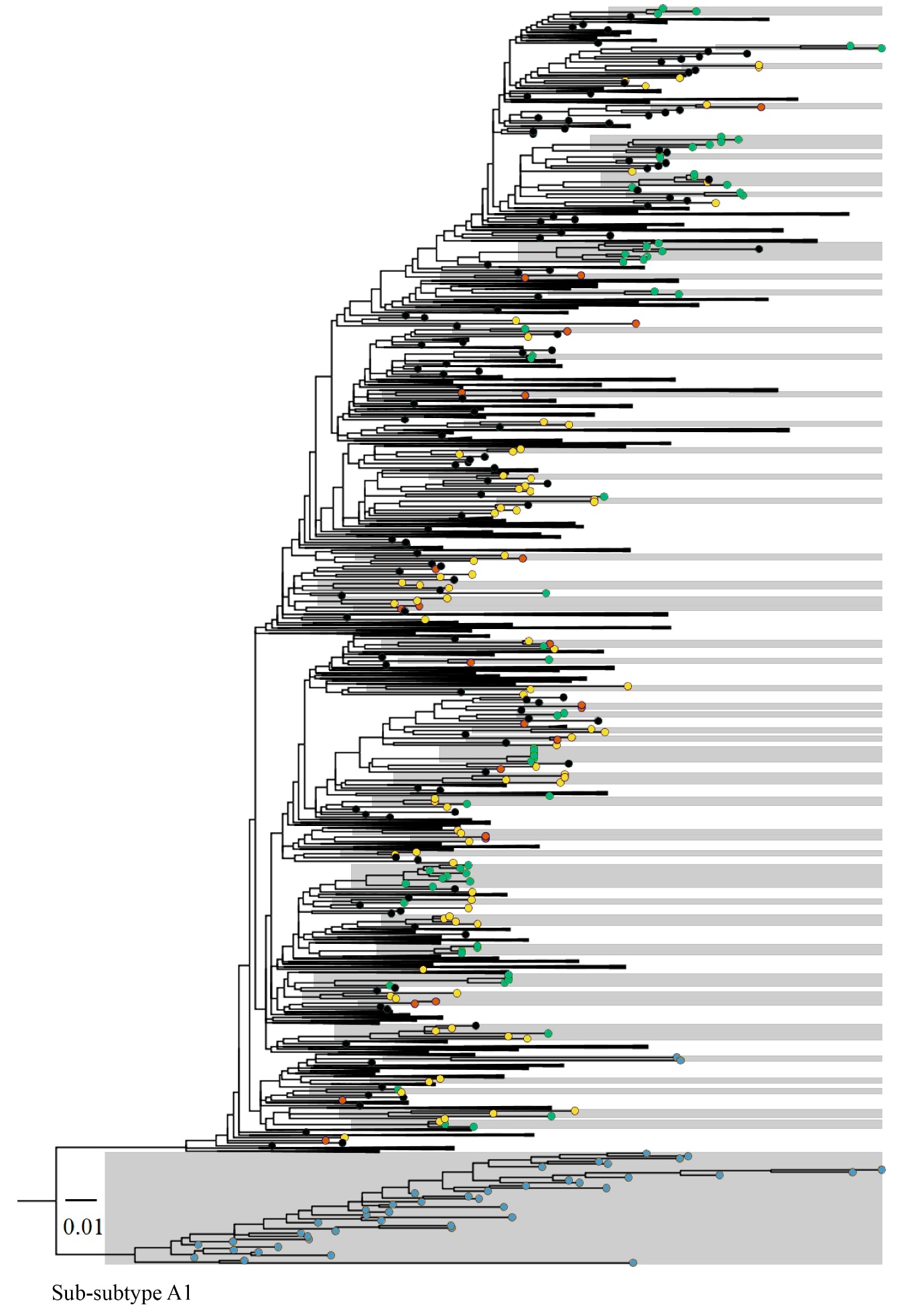

2.
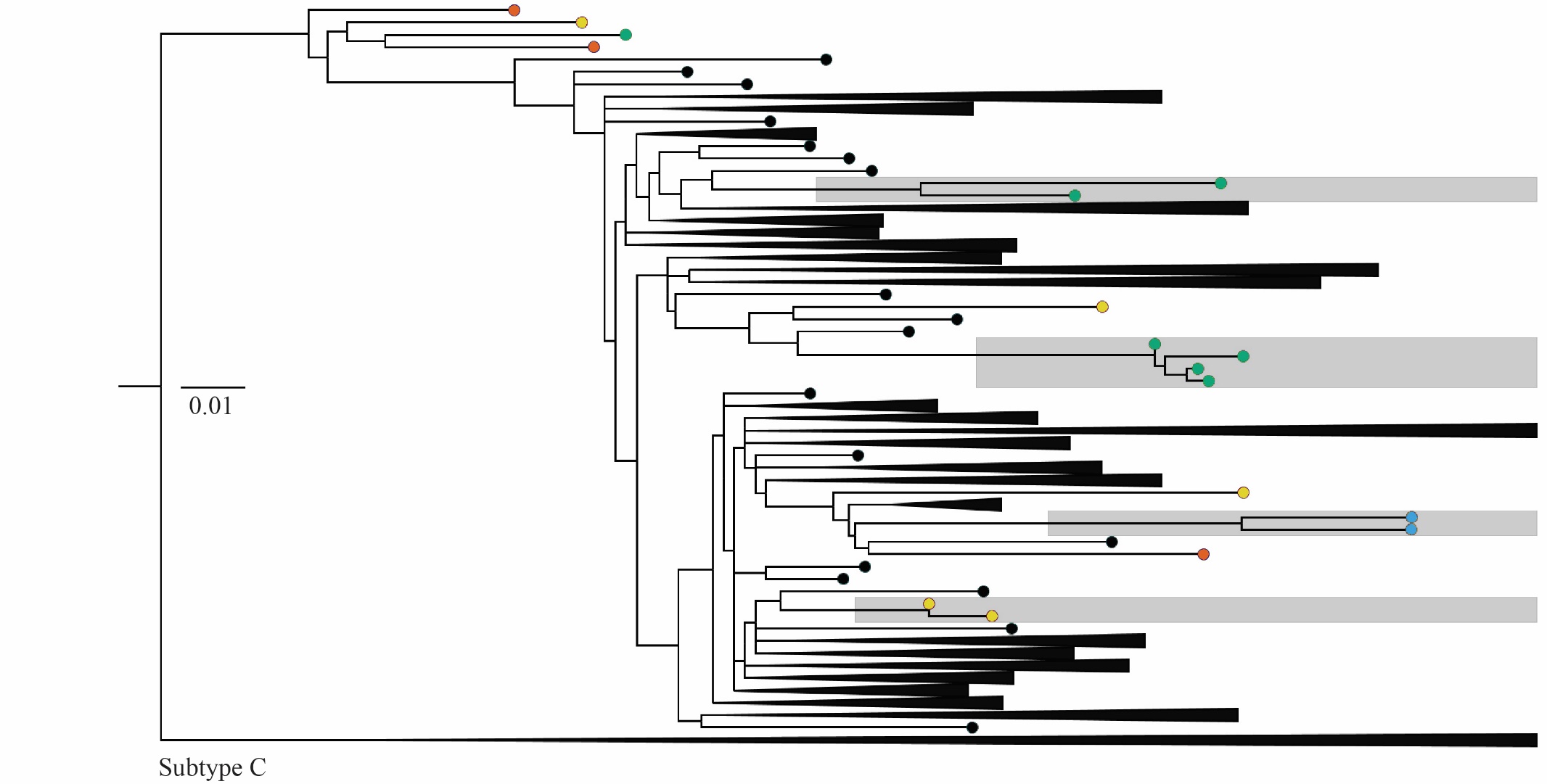

3.
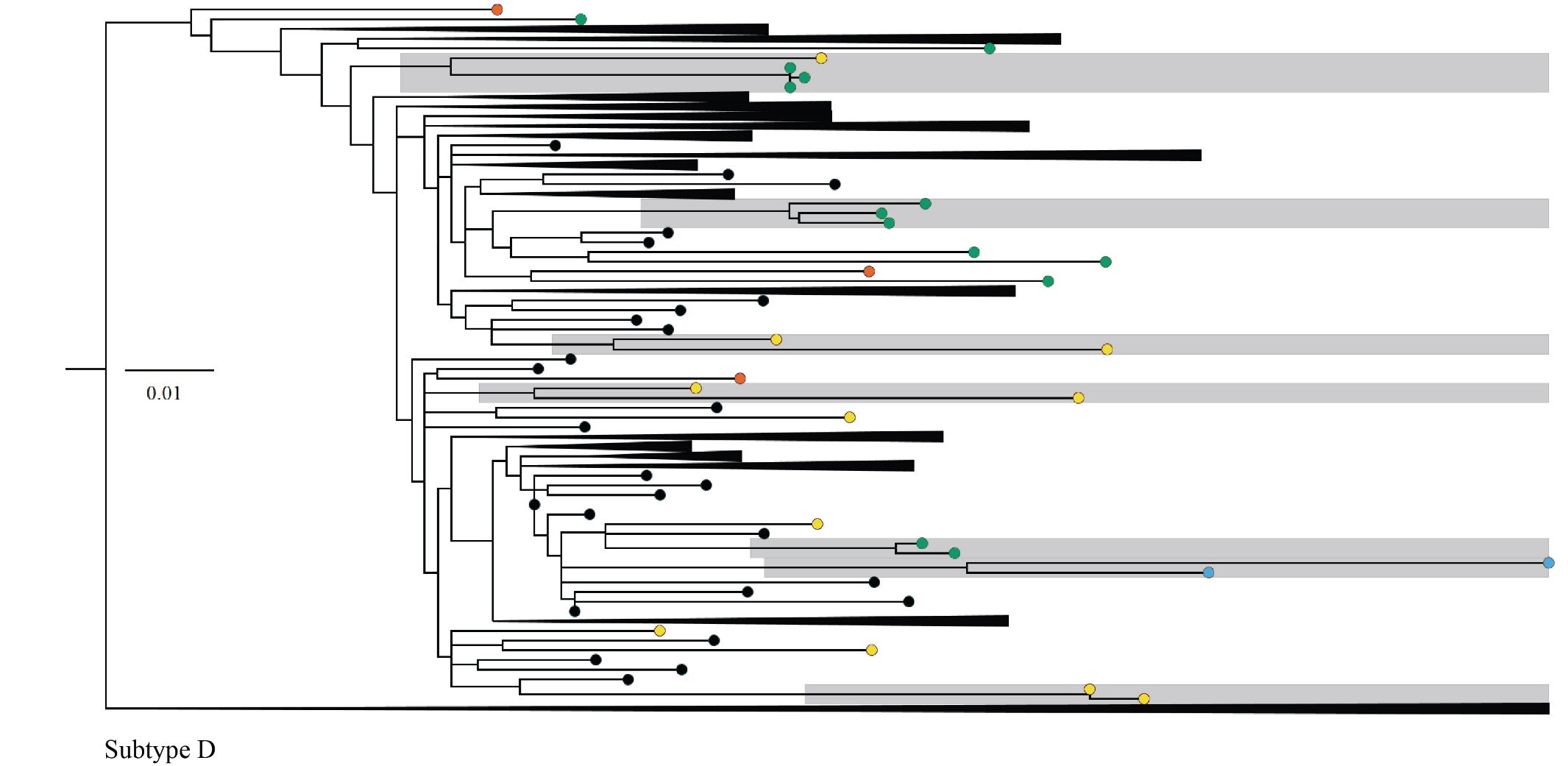


## **Figure S3**

## **
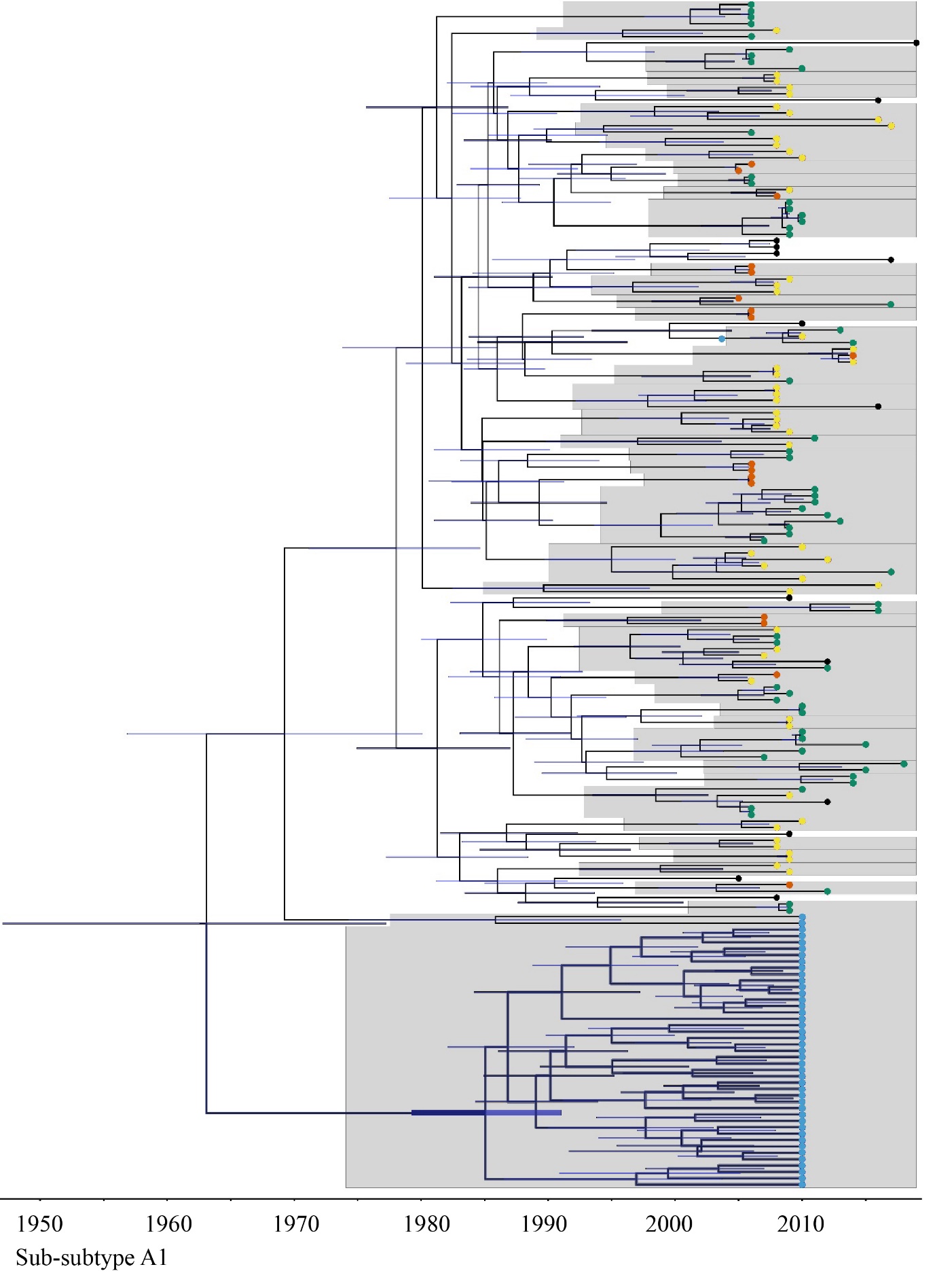
**

1.
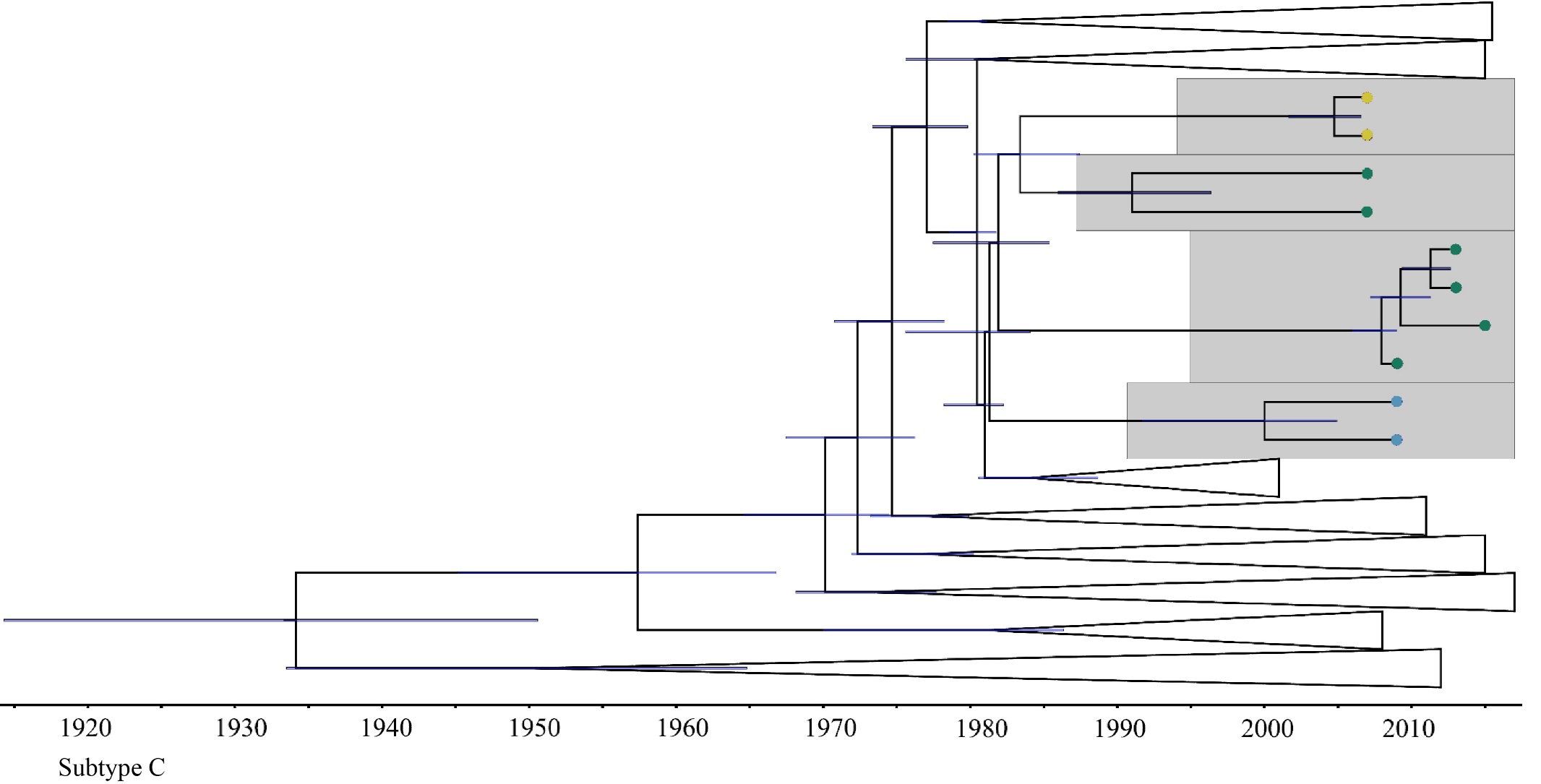

2.
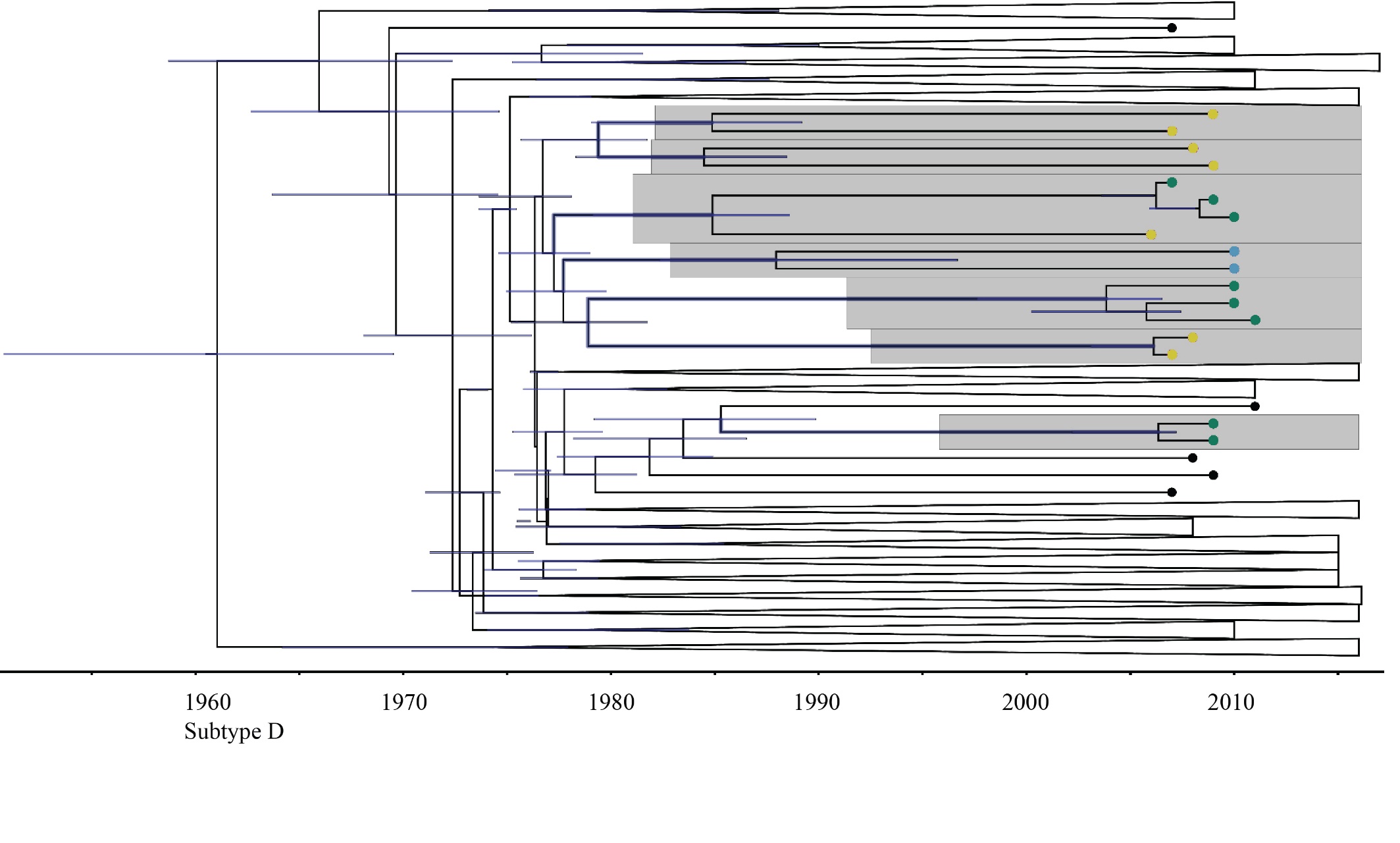


**Supplementary references**

1 National AIDS and STI Control Programme. *Kenya HIV County Profiles 2016.*, <<http://nacc.or.ke/wp-content/uploads/2016/12/Kenya-HIV-County-Profiles-2016.pdf>> (2017).

2 Kenya National AIDS Control Council. *Kenya AIDS Strategic Framework 2014/2015–2018/2019*, <<http://nacc.or.ke/wp-content/uploads/2015/09/KASF_Final.pdf>> (2019).
